# Transition from parents to caregivers of a child with type 1 Diabetes Mellitus: a scoping review

**DOI:** 10.1590/0034-7167-2022-0201

**Published:** 2023-01-30

**Authors:** Fábio Alexandre Melo do Rego Sousa, Maria de Lurdes Monteiro Serrabulho Andrade, Célia Maria Gonçalves Simão de Oliveira

**Affiliations:** I Unidade de Saúde da Ilha de São Miguel. Ponta Delgada, São Miguel-Açores, Portugal; II Associação Protectora dos Diabéticos de Portugal. Lisboa, Portugal; III Escola Superior de Enfermagem de Lisboa. Lisboa, Portugal

**Keywords:** Diabetes Mellitus, Type 1, Parents, Parenting, Child Care, Review Literature as Topic

## Abstract

**Objectives:**

to map and summarize the existing scientific evidence on parents’ transition experience to exercise the caregiver role of a child with 1DM, identifying gaps in knowledge of this experience.

**Methods:**

a scoping review was carried out based on JBI methodology, in two databases, following the Preferred Reporting Items for Systematic Reviews and Meta-Analyses extension for Scoping Reviews checklist.

**Results:**

we included 31 articles. From the studies, constitutive elements of parents’ transition experience to caregiver role of a child with 1DM were found, which focused on the nature of the experience, the feelings and emotions experienced, the hindering conditions, the facilitating conditions, the strategies used by parents and the results or effects obtained.

**Final considerations:**

the transition process’ characterizing elements were identified, but not a theoretical explanation of it. Additional research should be carried out in order to allow a deeper understanding of this process.

## INTRODUCTION

Type 1 Diabetes Mellitus (1DM) is usually diagnosed during childhood or adolescence, becoming the most common chronic metabolic disease in pediatric age^(1-2)^. The number of children and adolescents with diabetes increases every year, and in populations of European origin, almost all children and adolescents with diabetes have type 1. It is estimated that more than 98,200 children and adolescents under the age of 15 are diagnosed with Diabetes Mellitus annually, and this number increases to 128,900 when the age group extends to 20 years^(3)^.

In 1DM, due to children’s and some adolescents’ inability to take responsibility for the self-management of their chronic condition, this function falls on parents and, in particular, on mothers^(4-7)^. It is socially expected that taking care of a child is carried out as an exercise inherent to the role of being a parent of any child, i.e., something intrinsic to the parental role. However, the exercise of this role acquires an additional responsibility, when children have a certain health condition that imposes functional limitations or when a long-term dependence is expected^(8)^. Thus, becoming a caregiver for a child with a long-term chronic condition introduces an additional role, requiring a reorganization of priorities and a redirection of energy by caregivers^(8)^. The experience of this complex situation has important and potentially negative consequences if health services and social support systems are unable to help these caregivers. Being a caregiver of a child with a chronic disease is connoted to a dynamic process, in which an individual goes through a sequence of steps, requiring considerable transitions and restructuring of responsibility over time^(8)^.

In 1DM, we are faced with a situation of special complexity in which parents are required to additionally deal with the transition to the role of caregiver for a child with a chronic illness that requires a response that cannot be postponed and that is expected to be adaptive, promoting stability and well-being for all family members.

Chick & Meleis defined the concept of transition as the passage from one phase of life, condition or stable state to another, being a process triggered by a change^(9-10)^. Transition, as a process and result, emerges from the complex network of interactions between person-environment, which may involve more than one person, and is integrated in a given context and situation, denoting a change in the health status or in the relationships of roles, expectations or skills^(9-10)^. This concept is considered by Meleis as congruent or related to other concepts essential to this problem, such as those of adaptation, adjustment and self-care^(11)^.

1DM diagnosis to a child is an impacting experience capable of altering parental and family dynamics, leading to the installation of an initial “chaos” that emerges from the vulnerability felt by parents regarding the health situation of their children, as well as the complexity of care that they have to take and the changes that this situation can trigger in their own processes and life expectations. Parents are presented with a unique set of challenges that constantly appeal to their ability to adapt, organize and care. Knowing how these parents’ transition to the role of caregiver is characterized is relevant for nursing care so that this can be a facilitating factor for a healthy transition.

## OBJECTIVES

To map and summarize the existing scientific evidence on parents’ transition experience to exercise the caregiver role of a child with 1DM, identifying gaps in knowledge of this experience.

## METHODS

### Study design

The review was guided by the methodology proposed by the JBI for scoping reviews^(12)^. The choice for this type of review was based on its recognized usefulness, when it is intended to map and summarize scientific evidence, allowing the analysis of how research on a particular phenomenon has been conducted, as well as identifying gaps in existing evidence, pointing to the need for additional research^(13-14)^.

### Methodological procedure

A previous systematic review protocol (unpublished) was elaborated, adopting the Preferred Reporting Items for Systematic Reviews and Meta-Analyses extension for Scoping Reviews (PRISMA-ScR)^(15)^. To develop this review, we followed the JBI Manual (version 2020)^(12)^ recommendations, which includes the following steps: defining title, question and research objectives; defining inclusion criteria; defining strategy for research and selection of evidence sources, for data extraction, analysis and summary of evidence and for presentation of results.

Using the Participants, Concept and Context (PCC)^(12)^ strategy, studies were included in the review in which participants (P) were parents (mothers and/or fathers) of children and adolescents diagnosed with 1DM, or another primary family caregiver of children. Studies in which children with 1DM were diagnosed with another chronic disease were excluded, in order not to lose the specificity of the topic of this review. Regarding the concept (C), studies that addressed parents’ transition experience to exercise the role of caregiver of a child with 1DM were included. As for the context (C), and considering the objective of this review, no specific one was identified. Based on this strategy, this review sought to answer the following question: what is parents’ transition experience to exercise the role of caregiver of a child with 1DM?

We included in the review all primary (qualitative, quantitative and mixed) and secondary (literature reviews of different types, including meta-analyses or meta-synthesis) studies that related to the review question and fulfilled the defined inclusion criteria. We also look for unpublished studies in the digital repositories of libraries of Portuguese higher education institutions, in the CAPES theses database (Brazil), in the Portugal Open Access Scientific Repository (RCAAP - *Repositório Científico de Acesso Aberto de Portugal*) and in the OpenGrey database.

### Data source and search strategy

The defined search strategy intended to identify published and unpublished studies until February 2020, and the period for selecting articles took place from February to July 2020. The searches were conducted electronically and manually, the latter being carried out by searching for relevant articles in the bibliographic reference listings of selected articles. As recommended by JBI^(12)^, the three-step research strategy was adopted. The first step involved carrying out an initial search carried out in CINAHL Complete (via EBSCO) and in MEDLINE Complete (via PubMed), using keywords and preliminary search expressions obtained from natural language: “Diabetes”, “Type 1 Diabetes”, “Child*”, “Infant”, “Pediatric”, “Adolescen*”, “Parents”, “Mother*”, “Father*”, Family caregiver*”, “Transition*”, “Parent* Experience”, “Adaptation”, “Coping” and “Parent* adjustment”. Then, we analyzed the words used in the titles and abstracts of obtained articles, in order to identify relevant additional terms, as well as indexing terms used by the selected databases (Subject Headings, in the case of CINAHL, and MeSH terms, in the case of MEDLINE), to describe the articles. The PCC elements, previously presented, were articulated to define the search terms and their combinations, and several tests were carried out in order to obtain research strategy refinement.

The second stage comprised a new search carried out, separately, in the CINAHL Complete (via EBSCO) and MEDLINE Complete (via PubMed) databases from the search terms in natural language and the indexing terms identified in the previous step, using the Boolean operators «OR» and «AND» (Chart 1).

**Chart 1:** Terms used in search strategy

	Natural language	CINAHL complete (Subject Headings)	MEDLINE complete (MeSH)
P (population)	(Type 1 Diabetes) **AND** (Child^*^ **OR** Infant **OR** Pediatric **OR** Adolescen^*^) **AND** (Parents **OR** Mother^*^ **OR** Father^*^ **OR** Family caregiver^*^)	(Diabetes Mellitus, Type 1) **AND** (Infant **OR** Child **OR** Adolescen^*^ **OR** Pediatric) **AND** (Parents **OR** Mothers **OR** Fathers **OR** Caregivers)	(Diabetes Mellitus, Type 1) **AND** (Infant **OR** Child **OR** Child, preschool **OR** Adolescent) **AND** (Parents **OR** Mothers **OR** Fathers **OR** Caregivers)
C (concept)	(Transition^*^ **OR** Parent^*^ Experience **OR** Coping **OR** Adaptation **OR** Parent^*^ adjustment)	(Coping **OR** Health transition **OR** Adaptation psychological **OR** Life change events **OR** Parenting	(Adaptation psychological **OR** Emotional Adjustment **OR** Parenting **OR** Life change events)
C (context)	Not defined	Not defined	Not defined

Chart 2 presents an example of the research strategy used in the CINAHL Complete database (via EBSCO) (research carried out on July 18, 2020).

**Chart 2:** Search strategy used in the CINAHL Complete database (via EBSCO)

Step	Search strategy	Results
S1	Diabetes Mellitus, Type 1	26,796
S2	Infant OR Child OR Adolescen^*^ OR Pediatric	1,281,304
S3	Parents OR Mothers OR Fathers OR Caregivers	305,018
S4	S1 AND S2 AND S3	2,189
S5	Coping OR Health transition OR Adaptation psychological OR Life change events OR Parenting OR Parent^*^adjustment OR Parent^*^ Experience OR Transition)	148.252
S6	S4 AND S5	413
S7	S4 AND S5. Limiters - date of publication: 20200231.	413

In the third step, the lists of bibliographic references of selected articles were analyzed, and other relevant studies were identified. The extracted material was imported and managed through the bibliographic reference management software Mendeley^©^, and the duplicate sources were eliminated. Each article’s title and abstract were read in full, and those that met the defined inclusion criteria were selected. Whenever doubts arose about the relevance of the study from the abstracts, the full article was retrieved. Study selection was performed by two reviewers. The cases in which there were doubts or divergences were resolved with the intervention of a third reviewer.

### Data extraction

Data were extracted using the extraction instrument developed and agreed upon by the researchers, which included as items the identification of authors, year and country of origin, information about participants, methodology used and main results found. The data extraction process was carried out by two reviewers, and differences that arose were resolved through discussion between them, with no need to resort to a third reviewer.

Considering the objectives and the research question, it was decided to summarize the main results of included studies through descriptive qualitative content analysis^(12,16)^. The results of this review are presented in Charts 3 and 4.

**Chart 3:** Identification, characterization and presentation of the main results of included studies

Authors	Year/country	Methodology	Sample (N)	Main results
Iversen, Graue, Haugstvedt & Råheim^(1)^	2018 Norway	Phenomenology	15 (8 mothers and 7 fathers)	It took a long time for parents to convince themselves that disease diagnosis had changed their lives forever. Familiarizing themselves with the new situation was a challenge, as they had to quickly learn about the disease and how to manage it. The establishment of strict routines for glycemic control was crucial. Fathers and mothers lived through a period of mourning and sadness. After 6 months to 1 year of diagnosis, parents reported being more familiar with the situation, however, they continued to be very concerned about their children’s quality of life and the possibility of future complications. Parents lived in a state of constant alertness and physical and mental readiness, feeling constantly exhausted. With the return to school, parents took on the responsibility of educating/training school staff on how to care for the child with 1DM.
Holmström, Häggström & Söderberg^(17)^	2018 Sweden	Qualitative	13 (10 mothers and 3 fathers)	The appearance of 1DM in children was experienced as a life change for parents and family. After diagnosis, health professionals helped parents feel safe, cared for and included in the responsibility of managing the therapeutic regimen. Parents learned more about the disease (through observation and questioning from professionals) so that they could control the change in their daily lives. It was difficult to learn how to inject insulin or monitor capillary glucose levels. With greater knowledge of the disease, parents realized that 1DM was a condition that required taking medication for the rest of their lives and being in constant control. After discharge, the parents had to take responsibility for managing the disease alone, and the basic knowledge that was transmitted in the hospital was not enough to manage the disease at home. Parents realized that good communication with the school was essential to ensure their children’s safety during the day, and they felt responsible for managing the illness even when they dropped their children off at school. 1DM management affected parents’ professional activities and many had to change jobs in order to gain more flexibility in supporting children during the school day.
Khandan, Abazari, Tirgari & Cheraghi^(18)^	2018 Iran	Phenomenology	11 mothers	In addition to performing the maternal role, mothers had to additionally be caregivers. To play this role, they had to face several challenges, acquire knowledge and skills, requiring family support. Capillary blood glucose monitoring and insulin administration were painful procedures, and children had difficulty accepting them, and mothers, performing them. Mothers turned to different sources of information to learn more about 1DM, diabetes association, libraries, the internet and other parents of children with 1DM. Mothers experienced feelings of guilt, anxiety and obsession, making it very difficult to manage the daily duties associated with managing the illness and meeting the children’s care needs. With 1DM diagnosis, mothers faced an acute emotional crisis. Mothers lived a life full of worries, knowing that the disease was a threat to their children’s health and that it could negatively affect them in the future.
Herbert, Wall, Monaghan & Streisand^(19)^	2017 United States	Randomized controlled study	134 (120 mothers and 14 fathers)	22% of parents reported having ceased and/or reduced their working time; 15%, although they continued to work, requested flexibility, in order to ensure responsibility for the therapeutic management of their children’s 1DM; 7% of parents suffered the negative impact of diabetes on their careers, including their professional performance and the opportunity for career progression.
Pierce, Aroian, Caldwell, Ross, Lee, Schifano, et al.^(20)^	2017 United States	Qualitative	162 (137 mothers and 25 fathers)	For parents, it was difficult to manage the 1DM therapeutic regimen of children aged five years or younger, as they did not understand the need to comply with such a regimen, nor did they identify or report a crisis. Children reacted negatively to food and activity restrictions and painful procedures. Parents felt tired of the constant surveillance and sleep deprivation, the lack of social support and the incessant focus on feeding their children. They regretted the loss of a more spontaneous lifestyle, felt isolated and expressed grief, guilt, anger and concern for the child’s future. Over time, parents adapted, achieving “a new normalcy”, a balance between “perfect” control and living as normally as possible. They used for coping: recognizing that the new technology has greatly improved child care and quality of life, enjoying unusual moments in which 1DM did not dominate, and being involved in educational and advocacy initiatives for 1DM. The constant focus on the therapeutic management of 1DM harmed couple intimacy, as they had difficulty spending time alone, because 1DM dominated conversations and interfered with sleep and exhaustion. Such a focus caused parents to devote less attention and less quality time to their other children. They reported great difficulty in integrating their children into babysitting programs or day care centers that accepted responsibility for the therapeutic management of 1DM. Almost all parents reported anguish in relinquishing the therapeutic regimen control, but gradually they learned to trust others. They mentioned work repercussions, such as accepting less demanding jobs, refusing promotions or choosing to work from home.
Rifshana, Breheny, Taylor & Ross^(4)^.	2017 New Zealand	Phenomenology	17 (14 mothers and 3 fathers)	The experience of caring for a child with 1DM was governed by a process of constant surveillance that required an attentive awareness, an active commitment, making the experience of caring a very tiring process. Planning the child’s routines was the most obvious strategy. Situating and comparing their children’s bodily experiences, in terms of other possible childhood conditions, allowed parents to manage some of the gains and losses of 1DM and to feel greater control over the situation, favoring their coping. These comparisons were used in two ways: favorably or unfavorably with respect to other child health conditions. 1DM has been described as a lifelong chronic condition with no cure, and there is no clear point from which parents stop grieving over the loss of their child’s health.
Pimentel, Targa & Scardoelli^(21)^	2017 Brazil	Qualitative	11 (9 mothers and 2 fathers)	1DM diagnosis was described as one of the most difficult moments in parents’ lives, being permeated by sadness, anguish, despair and suffering. The fear of the unknown, the disease therapeutic management/treatment and its prognosis led to difficulties in coping with unexpected situations arising from the need to perform care and apply instrumental skills. When well informed about the disease, parents and their children ended up creating adaptation mechanisms, and the feelings of fear, denial and despair ended up transforming into acceptance. After diagnosis, parents begin a new path towards adaptation, incorporating new life habits and a new routine to be followed. The lack of support and communication from health professionals, in particular nurses, was reported by parents, who felt a need for greater monitoring and clinical guidance from these professionals.
Rankin, Harden, Waugh, Noyes, Barnard & Lawton^(22)^	2016 United Kingdom	Qualitative	54 (38 mothers and 16 fathers)	In the first days after the diagnosis, all parents praised the clinical care provided to their children and the instructions given by professionals. They highlighted the difficulties in understanding and assimilating the information provided and the unknown medical terminology. Some felt stressed and extremely disturbed and, therefore, were unable to assimilate and retain the information and advice transmitted. They reported that the guidance provided by health professionals had not prepared them for how their lives would be affected, and several felt that professionals were not in the best position to provide empathic and nonjudgmental support. Many described traumatic experiences regarding the administration of insulin injections, having even physically persecuted and restricted the children. For fear of nocturnal hypoglycemia, not detected by the child, parents reported spending the night in a state of constant surveillance, with consequent exhaustion. Parents felt that early contact or home visits by professionals would have helped due to their particular circumstances. Peer support was experienced in opposite ways.
Boogerd, Maas-van Schaaijk, Noordam, Marks & Verhaak^(23)^	2015 The Netherlands	Qualitative	39 (29 mothers and 10 fathers)	Coping with diagnosis and complex disease management skills were described by parents as overwhelming, referring that feelings of uncertainty and anxiety were intensified when they went from training by professionals to self-management of regimen at home. 1DM and its management had a profound restrictive impact on parents’ social and work life and on children’s lives. Concerns about the consequences of 1DM in the short and long term generated stress and sadness. Adaptation to the disease and its treatment was difficult, and support from friends and family was not felt to be adequate, making the health team and parents of children with 1DM the main sources of support. Support by teachers and schools was variable. Parents’ skills for managing the therapeutic regimen increased as their child grew up. The health team’s availability allowed parents to feel supported and secure in caring for their children, however, it would benefit from being more individualized, aimed at decoding the various information available.
Lawton, Waugh, Noyes, Barnard, Harden, Bath, et al.^(24)^	2015 United Kingdom	Qualitative	54 (38 mothers and 16 fathers)	Some parents highlighted difficulties in understanding the information conveyed during their children’s consultations. Some professionals were praised for the clarity when conveying information, while others were criticized for using medical terminology, perceived as complex and difficult to understand. Almost all parents considered that participating in their children’s consultations was felt to induce stress and anxiety, because they feared being judged due to the possible deterioration of their children’s health, despite their efforts, conditioning the parents’ action.
Rodrigues^(25)^	2015 Portugal	Qualitative	10 (9 mothers and 1 father)	Parents reported insecurity in care by school staff, due to lack of knowledge, errors in the calculation of insulin doses and lack of interest in treatment. They mentioned concern and fear of being absent during a decompensation, insecurity in third-party care, fear of decompensation at night. They dealt with it being on permanent alert and changing family routines, depriving themselves of sleep and being absent from work. They reported difficulties in managing meal times, carbohydrate accounting and insulin dose calculations. They valued the health team’s support and the potential they could have in accompanying children and families at school and at home.
Ayala, Howe, Dumser, Buzby & Murphy^(26)^	2014 United States	Mixed study	63 fathers (Phase 4)	Fathers wanted health professionals to recognize living with 1DM as a dynamic process that evolves over time. They desired sensitivity and recognition of fathers’ unique experience and challenges. Fathers expressed anxiety about how to incorporate the basic requirements of diabetes into their daily lives. Using the skills learned in the hospital was a challenge. Upon returning home, fathers felt new for many months, depending on professionals. They were valued when professionals listened to them and understood that the guidelines given initially, in the hospital, had been little noticed, due to the emotional overload then experienced, and when professionals responded therapeutically to fathers’ grief, to their concern and uncertainty, taking time to understand their feelings in the struggle, to adapt to a new way of life. Fathers depended on professionals to take on a more directive role, when 1DM is out of control, in the face of a crisis or in the face of a phase of emotional overload.
Forsner, Berggren, Masaba, Ekbladh & Olinder^(27)^	2014 Sweden	Qualitative (longitudinal)	6 (3 mothers and 3 fathers)	After the diagnosis, parents felt that their lives had been turned upside down, leading to feelings of grief, anger and helplessness. Parents had to face their fears, obstacles and doubts, in particular about their ability to use the insulin pump. They reported feeling continually between a state of vulnerability and trust, between hope and despair, needing health professionals’ support. Over time, parents expressed relief in taking control of the disease therapeutic management, fully ensuring their parental role, caring for their child with greater security and less uncertainty. They consider that they have become specialists in their children’s diabetes and they verbalized feeling in control of the disease most of the time. However, there was a continuous pressure to always be aware of events, having a constant fear of losing control. Developing coping strategies, adjusting and, finally, accepting the new life after disease diagnosis meant for parents to reorganize their lives around their child’s needs. It implied giving up social life and accepting that they cannot improvise. Over time, 1DM and the insulin pump came to be considered as a natural part of family life.
Silva^(28)^	2014 Portugal	Qualitative (exploratory-descriptive, Phenomenological)	14 (10 mothers, 4 fathers)	1DM diagnosis changed the family dynamics, leading to a restructuring of life in terms of routines, medication, night care for the child and conditioning of social and work life. 1DM changed eating habits, led to greater surveillance and supervision of children and specific care (checking blood glucose and administering insulin). Parents sometimes constantly felt fear, insecurity, worry, sadness, anger and guilt, associated with the possibility of other pathologies and complications, hypoglycemia, adolescence and fear of making mistakes. Some siblings of children with diabetes felt jealous for the attention and affection given to this child. It was necessary to develop parental skills, such as learning about the disease and treatment and dealing with difficulties. Parents received support from health professionals, their own child with 1DM and family members, school and information on the internet.
Martins, Ataíde, Silva & Frota^(29)^	2013 Brazil	Qualitative	12 mothers	1DM diagnosis represented a change in mothers’ daily life, having a negative emotional impact, and the fear and anguish felt remained even after the diagnosis. Mothers’ non-acceptance of the disease interfered with their child’s well-being, causing stress and suffering. The level of attention and the differentiated care shown by mothers to their children with diabetes caused feelings of revolt and jealousy in the other children. Although 1DM affects the whole family, the responsibility for the therapeutic regimen care/management fell on mothers, and their intervention went beyond the instrumental care necessary for treatment.
Nurmi & Stieber-Roger^(30)^	2012 United States	Qualitative (collective case study)	13 (10 mothers and 3 fathers)	Paternity involved not only adjustments to accommodate 1DM-related tasks, but also changes in perspective and interactions with family, community, and society. Parents fought to affirm their children as “normal”, with a particular characteristic (having 1DM), more than a disease and to decentralize attention to diabetes, focusing on the children. Participating in fundraising, interacting with other children and families who also lived with diabetes, and sharing knowledge about the disease was considered important. The need for advocacy by children was felt, in particular, in the school context, where it was necessary to ensure that schools were prepared to deal with the particularities of daily life of a child with 1DM.
Dashiff, Riley, Abdullatif & Moreland^(31)^	2011 United States	Qualitative	40 fathers	Fathers found managing the therapeutic regimen difficult, with a sense of struggle (with the school system and with adolescents) and frustration marked by the need to be vigilant, evoking fear, sadness and worry. Some fathers exclusively reported negative feelings (guilt, sadness and fears) or positive feelings (relating to the conviction that they had done everything, even if it was not perfect), and some reported both.
Rearick, Sullivan-Bolyai, Bova, & Knafl^(32)^	2011 United States	Mixed study	21 (14 mothers and 7 fathers)	Having a “veteran” father in 1DM management who could be contacted was considered by parents as the most important aspect of the intervention, which made stressful situations more tolerable. Mentors shared with parents practical aspects related to 1DM management. Mentors often validated parents’ feelings, and helped them develop confidence in their daily management of the disease therapeutic regimen through sharing their experiences about the shock of diagnosis and interactions with health professionals, family members and friends.
Smaldone & Ritholz^(33)^	2011 United States	Qualitative	14 (7 mothers and 7 fathers)	Parents identified the team’s availability after 1DM diagnosis as an important factor of support. However, at home, they felt isolated in caring for their children, overwhelmed and full of doubts. Support groups helped to reduce this isolation felt, in particular, by mothers. Parental adaptation was more effective when the responsibility for decision-making about the disease was shared by parents. None of the parents considered that they had been able to completely master 1DM management.
Bowes, Lowes, Warner & Gregory^(34)^	2009 United Kingdom	Qualitative	17 (10 mothers and 7 fathers)	Parents described feelings of grief and guilt after diagnosis. Parents found the practical management of the therapeutic regimen difficult to handle at first, but over time they got used to the daily tasks they had to perform and accepted this routine as part of their lives. Triggers that induced recurrent feelings of sadness were identified, such as periods of hospitalization, the need for insulin injections, adolescence and the transition to adult health care. Management of the therapeutic regimen and knowledge of the consequences of poor control caused continued stress and anxiety, and parents remained concerned about their children’s future health. Parents recognized the need for emotional support, but most did not receive it from professionals. Emotions and feelings associated with grief (sadness, guilt and anger) were expressed by parents.
Paterson & Brewer^(35)^	2009 Canada	Qualitative	9 (6 mothers and 3 fathers)	Parents of adolescents with 1DM felt the need for social support, especially regarding the assumption of the main responsibility for managing the therapeutic regimen of adolescents. The process of transitioning responsibility for managing the therapeutic regimen from parents to adolescent children was difficult, and conflicts were frequent. Parents felt the need for support, which should be provided by diabetes experts and by parents with adolescents with diabetes.
Athaseri, Tilokskulchai, Patoomwan, Grey, Knafl & Suprasongsin^(36)^	2008 Thailand	Qualitative	22 mothers	Maintaining normal capillary blood glucose levels and promoting the child’s independence were the mothers’ goals. Regarding 1DM management, the behaviors mentioned by mothers, in order to promote disease management and incorporate them into lifestyle, were administering insulin injections, testing capillary blood glucose, encouraging children to exercise, and taking children to follow-up medical appointments. Mothers played maternal roles, such as encouraging self-care, maintaining children’s identity, meeting developmental needs, identifying social support, resorting to alternative medicines, accepting their children’s illness, and developing coping strategies in the face of daily stress. Mothers mentioned skills training, close supervision, as well as maternal advice as strategies to promote and encourage their children’s independence and autonomy. In order to seek support and help, mothers essentially turned to health professionals, teachers and friends and family. The coping strategies used by mothers to manage daily stressors and reduce stress were participation in religious ceremonies, being patient, moving on and “stop thinking”. Mothers reported that husbands became more affectionate and closer after 1DM diagnosis. However, in other situations, conflicts arose between parents, due to disagreements about how to care for children with 1DM.
Sullivan-Bolyai, Rosenberg & Bayard^(37)^	2006 United States	Qualitative	14 fathers	The diagnosis was described as “shock and awe”, but fathers had to react quickly to the situation, learning to manage the illness and quickly takin responsibility. They used the “partnership in care” between fathers and wives, especially in problem solving and decision-making related to the disease therapeutic regimen, but also in the division of care tasks. Fathers used a healthy mantra for living with the disease: taking care of the child first and 1DM second, ensuring that the children with 1DM were treated like any other child.
Lowes, Gregory & Lyne^(38)^	2005 United Kingdom	Qualitative (longitudinal)	38 fathers	Twelve months after diagnosis, many fathers continued to be aware of their healthy child’s losses, control, freedom, spontaneity, confidence in their ability to protect their child from danger, and a sense of security. Fathers were aware of the risk of future complications. Initially, fathers were shocked and distressed, making it difficult for them to assimilate the complexity of the diagnosis. The need for insulin injections was not part of their family routine, leading to them having to carefully plan family activities and routines that were previously performed spontaneously. After diagnosis, fathers experienced anxiety and loss of confidence in their ability to deal with the situation. As their knowledge increased, they became more confident in 1DM management, but they continued to feel insecure about their ability to keep their child safe. A hypoglycemic attack, with loss of consciousness, was the greatest of all fears. Events such as hypoglycemia, being sick, a new school, vacations and family outings constantly brought 1DM to the forefront. Fathers were afraid of the possibility of long-term complications and balanced their fears with optimism about the development of treatments and with a future cure. After 4 months, most fathers reported feeling adapted to the new routines and diagnosis. After 12 months, all fathers got used to the changes implemented, integrating 1DM into their daily lives.
Mellin, Neumark-Sztainer & Patterson^(39)^	2004 United States	Qualitative	32 (24 mothers, 7 fathers and 1 guardian)	Parents expressed concerns about the disease: fear of long-term complications; who would help their daughter to control hyper or hypoglycemia when she was away from home; the future departure of daughters from home; whether the 1DM management would be good enough; and the onset of hypoglycemic crises. 1DM made it more difficult for the family to leave. Coping behaviors used in 1DM management included: providing concrete support to daughters in meeting food and medical needs; organizing their family life and schedules to facilitate the therapeutic regimen management; monitoring and reminding daughters about diabetes-related tasks; communicating openly inside and outside home about their daughters’ illness; and maintaining normalcy within the family. Coping strategies used to reduce worries were maintaining a positive attitude, monitoring the daughters at night and changing lifestyle (moving from full-time to part-time jobs, giving up going out at night).
Lowes, Lyne & Gregory^(40)^	2004 United States	Qualitative	38	The diagnosis made parents feel sad and in shock, initially trying to deal with the disease, focusing on the practical aspects of its management and prioritizing their children’s immediate needs. The information provided was a lot, and they had to assimilate little by little. Fear of hypoglycemia was frequent and, for some, it was difficult to allow their children the level of freedom they previously enjoyed. Coping strategies used were “live one day at a time”, establish a routine, “plan ahead” and talk to others with knowledge about diabetes. Professional help was important for parents. Mothers, 6 to 8 weeks after diagnosis, experienced acute distress and a feeling of continuous loss. Changes in eating routines included adapting meal times to diet management, school meals and children’s parties. Parents described their children’s illness as “a way of life”, with established routines integrated into their days. Twelve months later, parents began to consider diabetes part of their daily lives, with the children’s needs being incorporated into their lifestyle. Nocturnal hypoglycemia was a concern of parents, who continued to feel a loss of spontaneity and the need to constantly plan every aspect of their lives.
Sullivan-Bolyai, Deatrick, Gruppuso, Tamborlane & Grey^(41)^	2003 United States	Qualitative	28 mothers	Mothers used hypervigilant behavior on a daily basis as a way to manage their children’s 1DM. Three dimensions of constant surveillance were identified: day-to-day concerns; the daily management of 1DM; and internal and external support resources. In the first six months after diagnosis, mothers felt isolated and incompetent in the care they provided and in therapy management. Over time, they felt more prepared and competent in disease management and in assessment and interpretation of their own behaviors manifested by their children in the face of 1DM.
Sullivan-Bolyai S, Knafl, Deatrick & Grey^(42)^	2003 United States	Mixed study	28 mothers	The target behavior focused by all mothers was “glycemic control”, together with the daily disease therapeutic regimen, which was characterized as “constant surveillance”. Mothers used strict compliance as an approach to managing child care, essentially in the initial months after diagnosis, which implied frequent telephone contacts and consultations with the nurses responsible for 1DM education. Regarding flexible compliance, mothers reported feeling more competent and confident over time, and it took from 6 months to 1 year after diagnosis for them to feel confident. As mothers built up their level of confidence, they tended to adopt a more flexible treatment compliance, adapting the therapeutic regimen to make it easier to deal with.
Seppänen, Kyngäs & Nikkonen^(43)^	1999 Finland	Qualitative (Case study)	4 (2 mothers and 2 fathers)	The process of parental coping with 1DM included six phases: disbelief; lack of information and guilt; learning to care; normalization; uncertainty; and reorganization. The first three phases of the coping process took place in the hospital. The fourth phase described parental coping after child discharge. The fifth and sixth phases were related to children’s self-care at home during the first 2 weeks after discharge.
Faulkner^(44)^	1996 United States	Qualitative	27 (7 mothers and 7 fathers)	Regarding the effects caused by 1DM in the family, the parental responses were: remembering the diagnosis; changing the diet; predicting/scheduling daily activities; coping; and managing the therapeutic regimen. Regarding family roles, it was possible to identify characteristic roles taken by mothers and others by fathers. Teaching one’s own children, teachers, classmates and other parents about diabetes has been incorporated into the maternal role.
Hatton, Canam, Thorne & Hughes^(45)^	1995 Canada	Qualitative (Interpretive with phenomenological approach)	8 (8 mothers and 8 fathers)	Parents identified factors responsible for increased stress, such as the lability of children’s condition, the demands of complex management of the therapeutic regimen, painful procedures, multiple losses in children’s and family’s life, social isolation, inability to trust care for children and sharing feelings and anticipated concern with threats to their children’s safety, survival and future. The experiences of caring for children with diabetes were described in three phases: i) Diagnosis and hospitalization: the greatest stressors for parents were the severity of the clinical situation, causing fear of death and hospitalization, often terrifying, due to constant monitoring and painful procedures. Lack of support, associated with multiple stressors, generated common emotional responses, such as shock, anger, fear, grief, guilt, sadness, exhaustion, blaming others and themselves, feeling totally crushed, feeling desperate, hopeless, inadequate as parents, and feeling out of control. They used coping strategies: being assertive and defending the children; dealing with their needs; realizing that the complex therapeutic regimen has to be fulfilled for the sake of children’s life; seek help (hospital professionals); accepting the lack of support from fearful friends and family; and clinging to the hope of a cure in the future; ii) Taking care of children at home: bringing children back home and facing the responsibility for managing a chronic illness was the greatest stressor for parents. No longer having the hospital support system made them feel vulnerable and scared. The unpredictability of blood glucose fluctuations and the inability to anticipate and control them generated stress and frustration. The coping strategy used was to be totally vigilant and methodical with the 1DM therapeutic regimen. This intense learning phase lasted about 6 to 8 months after diagnosis and continued until a sense of adaptation was achieved; iii) Long-term adaptation: a phase of greater relaxation, leading parents to feel more in control of the situation, being able to incorporate the disease into family life in a long-term future. The main stressors identified by parents were related to children’s growth and development. Parents continued to feel the need for strategic coping centered on taking control, trusting others, and building support systems.

**Chart 4:** Characterization of parents’ transition experience to the role of caregiver of a child with Diabetes Mellitus 1

Categories	Characterizing elements of the transition experience
The nature of the experience	Six-phase parental coping process^(43)^
	Three-phase care experiences^(45)^
	Dynamic path “over time”^(1,20,26-27,34,38,40-42)^
Feelings and emotions experienced	Grief/losses^(1,4,20-21,27,31,34,38,40,45)^
	Sadness, shock, guilt, distress, anxiety, anger, obsession, powerlessness, and fear^(1,18,20-21,23-29,31,34,37-38,40,45)^
	Constant and diverse concerns^(1,18,20,22,25,28,31,34,38-39,40,45)^
	Between hope and despair, trust and vulnerability^(27)^
	Optimism^(38)^
	Positive and mixed feelings^(31)^
	Relief^(27)^
Hindering conditions/stressors	Facing treatment responsibility after hospital discharge^(23,45)^
	Insufficient information transmitted in the hospital^(17,22)^ and difficulty in its understanding and assimilation^(22,24,26)^
	Difficulty in managing care, meeting children’s needs at home^(17-18,21-23,25-27,31,34)^ and performing painful procedures^(18,45)^
	Risk of hypoglycemia ^(28,38-40)^, in particular the nocturnal^(22,25,40)^
	Insecurity in their ability to keep their child safe^(38)^ being cared for by others^(25,45)^ and at school^(25)^
	Inadequate support from friends and family^(23)^
	Unpredictability of blood glucose and inability to control it^(45)^
	Lack of social support^(20)^, health professionals^(21,34)^, peers^(22)^ and daycare/schools^(20,25,31)^
	School change, vacation, family outings^(38)^, parties^(40)^ and care context transition^(34)^
	Fear judgment by health professionals^(24)^
	Social isolation^(45)^
	Early age of children^(20)^, child growth and development^(45)^, adolescence^(35)^
	Children’s negative reaction to the therapeutic regimen^(20)^
Facilitating conditions	Health professional support (team)^(17,22-23,25-28,33,36,40)^
	Family support^(18,28,36)^
	Support provided by other parents with children with 1DM/support groups^(22-23,30,32-33,40)^
	Support provided by teachers and friends^(36)^
Strategies used by parents	Learning^(1,17-18,28,40)^ and developing skills on 1DM and therapeutic regimen management^(1,17-18,21,23,27-28,32,34,36-42,45)^
	Focusing on the practical aspects of managing 1DM and children’s needs^(27,36,39-40,45)^
	Intensely watching their children^(20,22,28,31,36,41-42,45)^ and being permanently alert^(1,25)^, even at night^(22,39)^
	Establishing routines for the children and family^(1,4,25,38-40,42,45)^
	Being methodical in the therapeutic regimen execution^(42,45)^ and searching in documentary sources^(18,28)^
	Changing children’s and family’s habits^(20,25,28,40)^
	Performing specific 1DM technical care^(25,27-28,36,38)^
	Managing the therapeutic regimen with progressive flexibility^(42)^
	Educating school staff^(1,44)^, engaging in educational and child advocacy initiatives^(20,30-31,44)^
	Struggling to affirm the children as “normal”, with a particular characteristic (having 1DM)^(30,37)^
	Seeking support of health professionals^(26,36,39,42,45)^, teachers, friends, family and other^(18,36,40)^ parents of children with 1DM^(18,32)^
	Taking responsibility and tasks: shared among the couple^(33,37,44)^ or care was performed by mothers only^(29)^
	Abdicating or reducing social life^(27,39)^
	Work adjustments: making schedules more flexible^(23)^, changing jobs^(17)^, reducing working time^(19,39)^, being away from work^(25)^, or home officing^(20)^
	Using alternative medicines^(36)^, participating in religious ceremonies^(36)^
	Recognizing the contributions of technology in the therapeutic regimen management^(20)^
	Comparing 1DM with other chronic conditions^(4)^, hoping for a future cure for the disease^(38-39,45)^
	Living one day at a time^(40)^, maintaining a positive attitude^(39)^, being patient, “moving on”, “stopping thinking”^(36)^, being assertive^(45)^ and communicating openly about the disease^(39)^
	Promoting and encouraging children’s independence and autonomy^(36)^
Results or effects obtained	Non-acceptance^(29)^ or acceptance^(21,27,34,36)^ of disease
	Awareness of permanent change in life^(1,4,17)^ and the need for strategic coping to take control, build support systems and learn to trust others^(45)^
	Exhaustion^(1,20,22,45)^, interference or sleep deprivation^(20,25)^
	Conflicts and losses in the relationships between parents due to disagreement on the way of caring^(36)^, conflicted relationship as a couple^(20)^ and conflicted relationship with the other children^(20,28-29)^, conflicts with adolescent children with 1DM^(35)^
	Greater closeness and affection of husbands after diagnosis^(36)^
	Negative impact on career/job^(19-20,23,28)^ and social life^(23,28)^
	Not being able to fully master 1DM management^(33)^
	Feeling more empowered to deal with the disease and treatment: feeling in control of the disease most of the time^(27)^, more prepared to deal with/manage the disease^(41)^, more competent, confident and flexible^(27,42)^, or safer in care^(23)^, integrating 1DM into family life as “new normalcy”^(20,34,38,40,45)^
	Learning to trust others^20^

## RESULTS

Through electronic search in databases, we identified 413 references in CINAHL Complete (via EBSCO), 449 in MEDLINE Complete (PubMed), 1 reference in the CAPES theses database (Brazil), 9 references in the RCAAP and 16 in the OpenGrey repository, totaling 888 records. Of these, 240 were excluded because they were duplicates, and 5 additional records were selected through reference lists of previously identified articles. Of the remaining 653 records, 511 were excluded after assessing title and abstract, and 3 were excluded because it was not possible to access their full text, even after efforts were made in this direction. After reading the full text of the 139 records, 108 were excluded because they did not meet the defined inclusion criteria, moving away from the topic under study (not focused on transition), because they integrated results common to parents and children (it was not possible to dissociate parents’ experience from that of children), or because they focused on marginal aspects of the transition experience under study.

**Figure 1:**
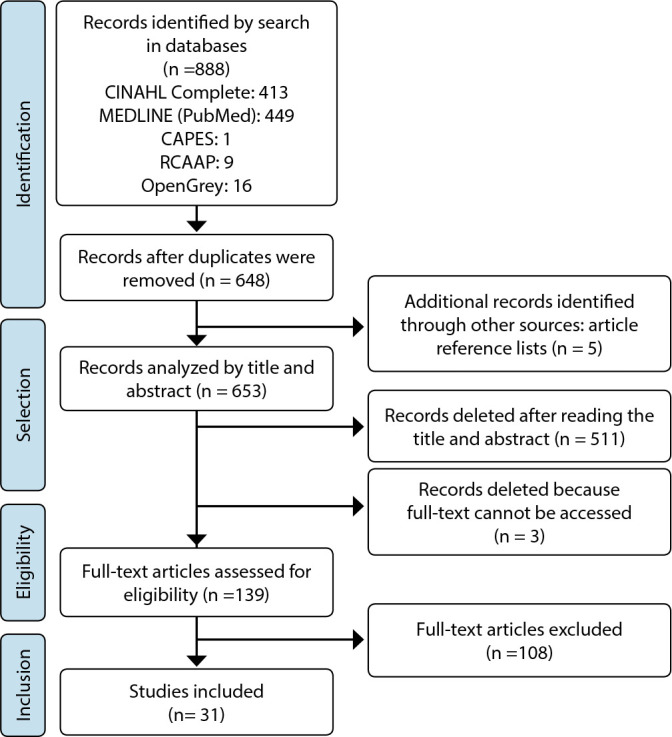
Study selection and inclusion process flowchart (PRISMA-ScR, 2018)

Finally, a total of 31 studies were included in this review (Figure 1). Regarding the period of publication, it was found that it ranged between 1995 and 2018. Regarding the country where the studies were produced, it was found that there was a wide geographic distribution, with 83.8% (N= 26) were performed in North America and Europe. Regarding sample composition, it was recorded that 27 included fathers and mothers, while 3 included only mothers and 1 only fathers. The primary articles’ results were read in full, and those related to the initial question formulated and the objectives of this systematic review were extracted (Chart 3).

Parents’ transition was summarized in six categories: the nature of the experience; feelings and emotions experienced; hindering conditions or stressors; facilitating conditions; strategies used by parents; and results or effects obtained (intermediate or final) (Chart 4). The textual elements that characterize that experience are also presented.

## DISCUSSION

From the results, it was possible to verify the existence of different studies that focused on parents’ experience in caring for children with 1DM. The nature of this experience implies a dynamic path that “occurs over time”^(1,20,26-27,34,38,40-42)^, tending to adapt to the situation of children’s illness and to achieve “a new normalcy” in personal and family life.

Only two studies identified and named the phases during the care experience: the phase of diagnosis and hospitalization; caring for children at home (after hospital discharge, facing responsibility for managing a chronic illness); and long-term adaptation^(45)^. In another study, it was possible to identify that the parental coping process was composed of different phases, such as disbelief, lack of information and guilt, learning to care, normalization, uncertainty and reorganization^(43)^.

The results of the studies are unanimous in considering that 1DM diagnosis triggered strong emotional responses in the parents and feelings of a predominantly negative tone, such as sadness, shock, guilt, anguish, anxiety, anger, obsession, impotence and fear^(1,18,20-21,23-29,31,34,37-38,40,45)^. However, at later times, some feelings of positive or mixed tint were also mentioned^(27,31,38)^. 1DM diagnosis was experienced by parents as a mourning, associated with different losses of a healthy child, freedom, spontaneity and confidence in the exercise of parental role^(1,4,20-21,27,31,34,38,40,45)^. The fear of the unknown, the disease therapeutic management and prognosis led parents to feel overwhelmed by the enormous responsibilities and unprepared to manage the disease’s complex therapeutic regimen.

Multiple concerns expressed by parents were identified, which were not limited only to the period of diagnosis, but that follow them throughout their lives, such as therapeutic regimen management^(31,34)^, hypoglycemia/decompensation^(25,40)^, hospitalization^(45)^, children’s leaving home^(39)^, threat to life^(18,45)^, possibility of future complications^(1,22,28,34,38-39)^ and those related to children’s future quality of life^(1,18,20)^.

From the evidence found, it was possible to recognize that the experience of transition to the role of caregiver of a child with 1DM was marked by multiple hindering conditions or stressors that parents had to face. Difficulties were identified in understanding and assimilating the information transmitted in the hospital about the disease and its management ^(22,24,26)^, and, in some situations, this was considered insufficient^(17,22)^. The feelings of uncertainty and anxiety were intensified when there was a transition from hospital care to self-manage the therapeutic regimen independently at home^(23)^. Bringing children back home and facing the responsibility for managing a chronic disease was a stressor identified^(23,45)^, as well as the difficulty in managing care, meeting their needs at home^(17-18,21-23,25-27,31,34)^ and performing painful procedures^(18,45)^, such as assessing capillary blood glucose and administering insulin. The risk of hypoglycemia^(28,38-40)^, particularly those occurring at night^(22,25,40)^, was another reported stressor. The unpredictability of blood glucose fluctuations and the difficulty in controlling them^(45)^ also contributed to the insecurity felt by parents, not only in their own ability to keep their child safe^(38)^, but also in the care provided by third parties^(25,45)^ or at school^(25)^.

The lack of support felt by parents was identified in some studies by society^(20)^, by health professionals^(21,34)^, by peers^(22)^ and by daycare centers/schools^(20,25,31)^. In other studies, the inadequate support provided by friends and family was reported as a difficulty^(23)^, reinforcing the express need for greater support (including emotional support) by health professionals^(22-23,26,34-35)^, by other parents of children/adolescents with 1DM^(35)^ and by family members^(18)^.

The evidence found also demonstrates that it was only possible for parents to deal with the disease and respond to the therapeutic regimen requirements through the presence of facilitating conditions, i.e., the availability of different sources of support, such as the health professionals responsible for child surveillance^(17,22-23,25-28,33,36,40)^, the family^(18,28,36)^, other parents with children with 1DM/support groups^(22-23,30,32-33,40)^ and teachers and friends^(36)^.

To face 1DM and manage the therapeutic regimen, parents resorted to different strategies, initially trying to deal with the disease, focusing on the practical aspects of 1DM management and prioritizing children’s immediate needs^(27,36,39-40,45)^. Familiarizing themselves with the new situation was a challenge, as parents had to quickly learn about the disease and develop skills for the therapeutic regimen management^(1,17-18,21,23,27-28,32,34,36-42,45)^. These skills included performing specific care in 1DM, such as assessing blood glucose, counting carbohydrates, dosing and administering insulin, and handling insulin pumps^(25,27-28,36,38)^. The diagnosis of 1DM forced parents to make changes in family life and establish routines for children and family^(1,4,25,38-40,42,45)^. Change in eating habits was one of the most evident^(25,28,40)^. Intensely watching the child^(20,22,28,31,36,41-42,45)^ and being permanently alert, physically and mentally ready^(1,25)^, even at night^(22,39)^, were other strategies used. In terms of employment, 1DM management implied that parents made work adaptations, which included making schedules more flexible^(23)^, reducing the time of work activity^(19,39)^, being absent from work^(25)^, changing jobs^(17)^ or home officing^(20)^, all with the aim of gaining greater flexibility and ensuring greater presence in child support.

As they experienced insecurity regarding the care provided to their children during the school period^(25)^, parents took the responsibility of ensuring the training of school staff ^(1,44)^, engaging in multiple educational and child advocacy initiatives^(20,30-31,44)^. Always striving to affirm children as “normal”, with a particular characteristic (having 1DM)^(30,37)^, parents sought the support of health professionals^(26,36,39,42,45)^, teachers, friends and family^(18,36,40)^, as well as other parents of children with 1DM^(18,32)^.

Results or consequences observed by parents during the performance of their role as caregivers of children with 1DM were identified in the literature, results that vary and even evolve according to the transition process dynamism. As a consequence, studies highlighted the negative impact on social life^(23,28)^ and conflicted relationships between parents, intimacy wise^(20)^, as well as the existence of conflicts due to disagreement about the way of caring for the child^(36)^, or conflicts with adolescents with 1DM^(35)^. However, in another study, it was found as a result the greater closeness and affection of husbands after diagnosis^(36)^. The effort and attention dedicated to 1DM management also affected the parents’ relationship with other children as a result of jealousy and anger felt, due to the fact that parents spend more time and devote more attention to children with 1DM^(20,28-29)^.

Parents became aware of the permanent change in their lives^(1,4,17)^, starting to consider diabetes as part of their daily life. Having a child with diabetes and incorporating their needs into their lifestyle and family became the new normalcy^(20,34,38,40,45)^. Parents, although feeling exhausted^(1,20,22,45)^ and tired due to constant vigilance and sleep deprivation^(20,25)^, were learning to master the 1DM therapeutic regimen demands, to better understand the disease symptoms and how to regulate their children’s blood glucose levels, going from novices to experts, feeling in control of the disease most of the time^(27)^. However, acceptance of the disease, despite having been achieved in some cases ^(21,27,34,36)^, in others, it was never verified^(29)^. Many parents sought to achieve a balance between “perfect” control and living as normally as possible^(20)^. Even in a phase of greater adaptation, in which parents felt more prepared to deal with/manage the disease^(41)^, more competent, confident, flexible^(27,42)^ and safer in providing care^(23)^, they continued to recognize the need for strategic coping to take control, build support systems and learn to trust others^(20,45)^.

### Study limitations

As a limitation, it is pointed out that it was not possible to have access to the full text of three identified articles. In addition to this, the fact that 83.8% of selected studies were carried out in North America and Northern Europe could constitute a limitation of this study, given the disparity and weight of each country’s unique cultural aspects as well as the responses provided/existing by social systems and health services that will certainly influence the way the transition process under study is experienced by parents.

### Contributions to nursing

Mapping and summary the existing scientific evidence allowed the characterization of parents’ experience of transition to the role of caregiver of children with 1DM. Although this summary, by its nature, does not allow to extract implications for practice, it leaves indications for professionals (nurses), either about the arduous and transforming experience that 1DM in children implies for parents, or about the need for timely training and persistent care.

## FINAL CONSIDERATIONS

This review allowed us to identify and summarize elements that characterize parents’ transition experience to exercise the role of caregiver of children with 1DM. Despite the fact that the constitutive elements of this experience are globally characterized in the literature and the predominance of qualitative studies included in this review, theorization that makes it possible to understand the process of transition of parents to the exercise of the role of caregiver needs specific investigation. Therefore, further studies need to be carried out in order to theorize this transition process, enabling a thorough understanding of it.
